# Levels of Predominant Intestinal Microorganisms in 1 Month-Old Full-Term Babies and Weight Gain during the First Year of Life

**DOI:** 10.3390/nu13072412

**Published:** 2021-07-14

**Authors:** Sonia González, Marta Selma-Royo, Silvia Arboleya, Cecilia Martínez-Costa, Gonzalo Solís, Marta Suárez, Nuria Fernández, Clara G. de los Reyes-Gavilán, Susana Díaz-Coto, Pablo Martínez-Camblor, Maria Carmen Collado, Miguel Gueimonde

**Affiliations:** 1 Department of Functional Biology, University of Oviedo, 33004 Oviedo, Spain; soniagsolares@uniovi.es; 2 Diet, Microbiota and Health Group, Instituto de Investigación Sanitaria del Principado de Asturias (ISPA), 33004 Oviedo, Spain; silvia.arboleya@ipla.csic.es (S.A.); nuriajmhd@gmail.com (N.F.); greyes_gavilan@ipla.csic.es (C.G.d.l.R.-G.); 3 Institute of Agrochemistry and Food Technology (IATA-CSIC), 46980 Paterna, Spain; mselma@iata.csic.es; 4 Department of Microbiology and Biochemistry of Dairy Products, Instituto de Productos Lácteos de Asturias (IPLA-CSIC), 33300, Asturias, Spain; 5 Department of Pediatrics, School of Medicine, University of Valencia, 46010 Valencia, Spain; cecilia.martinez@uv.es; 6 Pediatric Gastroenterology and Nutrition Section, INCLIVA Research Center, Hospital Clínico Universitario Valencia, 46010 Valencia, Spain; 7 Pediatrics Service, Hospital Universitario Central de Asturias, SESPA, 33004 Oviedo, Spain; gsolis@telefonica.net (G.S.); msr1070@hotmail.com (M.S.); 8 Pediatrics Research Group, Instituto de Investigación Sanitaria del Principado de Asturias (ISPA), 33004 Oviedo, Spain; 9 Pediatrics Service, Hospital de Cabueñes, SESPA, 33201 Gijón, Spain; 10 Department of Statistics, University of Oviedo, 33004 Oviedo, Spain; UO266718@uniovi.es; 11 Department of Anesthesiology, Geisel School of Medicine at Dartmouth, Dartmouth, NH 03756, USA; Pablo.Martinez-Camblor@hitchcock.org

**Keywords:** infants, microbiota, *Staphylococcus*, *Enterococcus*, *Bifidobacterium*, weight gain

## Abstract

The early life gut microbiota has been reported to be involved in neonatal weight gain and later infant growth. Therefore, this early microbiota may constitute a target for the promotion of healthy neonatal growth and development with potential consequences for later life. Unfortunately, we are still far from understanding the association between neonatal microbiota and weight gain and growth. In this context, we evaluated the relationship between early microbiota and weight in a cohort of full-term infants. The absolute levels of specific fecal microorganisms were determined in 88 vaginally delivered and 36 C-section-delivered full-term newborns at 1 month of age and their growth up to 12 months of age. We observed statistically significant associations between the levels of some early life gut microbes and infant weight gain during the first year of life. Classifying the infants into tertiles according to their *Staphylococcus* levels at 1 month of age allowed us to observe a significantly lower weight at 12 months of life in the C-section-delivered infants from the highest tertile. Univariate and multivariate models pointed out associations between the levels of some fecal microorganisms at 1 month of age and weight gain at 6 and 12 months. Interestingly, these associations were different in vaginally and C-section-delivered babies. A significant direct association between *Staphylococcus* and weight gain at 1 month of life was observed in vaginally delivered babies, whereas in C-section-delivered infants, lower *Bacteroides* levels at 1 month were associated with higher later weight gain (at 6 and 12 months). Our results indicate an association between the gut microbiota and weight gain in early life and highlight potential microbial predictors for later weight gain.

## 1. Introduction

From birth, and initially depending on the delivery mode, the neonatal gut is colonized by a rapidly diversifying microbiota, reaching an adult-type microbiota around 3–5 years of life. During early life, other perinatal factors, such as feeding practices, environment or antibiotic treatments, also contribute to shaping the microbiota development [1]. Current evidence supports the role of this early microbiota in promoting and maintaining a balanced immune response and adequate brain development and, subsequently, in the future health of the infant [2,3]. Induction of early microbiota alterations by antibiotics use has been linked to allergic diseases [4], obesity [5], risk of colorectal cancer [6] and other potential non-communicable diseases (NCDs) later in life [7,8]. These studies underline the importance of the early life microbiota as a key driver for adequate infant development and later health. Moreover, recent evidence indicates that altering this early microbiota may also have long-lasting effects on body weight and weight gain in childhood and on the later risk of obesity during adulthood [9,10,11,12,13]. Indeed, higher birth weight and rapid growth during early life have been linked to increased risk of overweight and obesity during childhood and adulthood [14,15,16,17,18]. Interestingly, a recent study reported the very early life microbiota which is present in meconium or first-pass neonatal samples as a predictor of infant overweight by the age of 2 years [19]. Early microbiota composition has also been linked to overweight and obesity at later infancy [20,21]. On the other side, other recent studies have highlighted the effect of antibiotic treatment on infant growth and development during the first 6 years of life [12].

In this context, the potential relationship between early microbiota and weight gain is of great interest since this relationship offers opportunities for the microbiota-mediated modulation of weight gain [22] and/or the prevention of growth impairment [23]. Recently, some studies have assessed the potential association between early microbiota and weight gain in preterm infants [24,25]; however, data on full-term babies are still scarce.

In this study, we evaluated the association between the early life microbiota and the later weight gain in both vaginally and C-section-delivered healthy full-term infants. We aimed at identifying if the levels of selected microorganisms at 1 month of age were related to later weight gain during the first year of life in these two groups of infants. With this goal, we used quantitative PCR (qPCR) for assessing specific microbial groups in the infant’s feces at the age of 1 month and monitored weight gain during the first year of life.

## 2. Subjects and Methods

### 2.1. Recruitment and Sampling

A total of 124 infants born after a full-term, uncomplicated pregnancy by vaginal delivery (*n* = 88) or by C-section (*n* = 36) were recruited at the neonatology units of the University Central Hospital of Asturias (Oviedo, Northern area, Spain) and the University Clinic Hospital of Valencia (Valencia, Mediterranean area, Spain). Inclusion criteria were no metabolic (obesity, diabetes) or chronic diseases and no probiotics consumption by mothers during late pregnancy or infants’ early life and no antibiotics administration to the infants. Clinical data such as gestational age or perinatal maternal antibiotics were recorded, as well as neonatal weight and height at birth, at 1, 6 and 12 months of life, the change in weight (weight gain), Z-scores for weight, height and weight-for-height were calculated for each infant at each time point. A fresh fecal sample was collected at 1 month of age and immediately frozen for later microbiota analyses.

Families received detailed study information and signed an informed consent form. The study recruitment and sampling have been approved by the Regional Ethics Committee of Clinical Research of Asturias (Ref. 12/16. 3 February 2016), the Ethics Committee of the Hospital Clínico Universitario de Valencia INCLIVA (Ref. 9 January 2015) and the Committee on Bioethics of CSIC. The procedures were performed in accordance with the fundamental principles set out in the Declaration of Helsinki, the Oviedo Bioethics Convention, the Council of Europe Convention on Human Rights and Biomedicine and the Spanish legislation on bioethics. The Directive 95/46/EC of the European Parliament and the Council of 24 October 1995, on the protection of individuals regarding the processing of personal data and on the free movement of such data were (and will be) strictly followed.

### 2.2. Fecal Microbiota Analyses

Total DNA was isolated from all fecal pellets as described previously by using the QiAGEN Stool Kit (QIAgen. Hilden. Germany) [26]. The extracted DNA was then used for quantifying fecal levels of the Enterobacteriaceae family, the *Bacteroides*-group and the genera *Bifidobacterium*, *Enterococcus*, *Staphylococcus* and *Lactobacillus* group by quantitative PCR using previously described primers, conditions and standard cultures (Table 1). PCRs were performed either in a LightCycler^®^ 480 Real-Time PCR System (Roche^®^) or a 7500 Fast Real-Time PCR system (Applied Biosystems) by using the SYBR Green. A subgroup of samples (*n* = 33) was analyzed in both machines to ensure comparability, without detecting statistically significant differences between the data obtained in each of them (data not shown), with both machines showing high correlation (Pearson’s correlation coefficients ranging between 0.785 and 0.942 depending in the primer pair used).

### 2.3. Anthropometrical Determinations

Child height and weight were recorded to the nearest 0.1  cm and 0.1  kg, respectively, through standardized procedures by pediatric nurse and registered at birth, 1, 6 and 12 months. With this information and the date of birth, Z-score was calculated by using WHO ANTHRO, Software for Calculating anthropometry, Version 3.2.2 (https://www.who.int/childgrowth/software/es/; accessed on 14 July 2021). The WHO Child Growth Standards provide child growth measures standardized by age and sex using Z-score.

### 2.4. Statistical Analyses

For statistical analysis, the free software R (https://www.r-project.org; accessed on 7 June 2021) was used. Variables are described by mean and standard deviations, median and percentiles or by counts and frequencies. Student–Welch and Chi-square tests were used for checking the mean and distributions equalities, respectively. Pearson correlation coefficients were used for studying the association between continuous variables. A heatmap, generated in R package using the heatmap.2 application in ggplots package [33] was employed for summarizing the analyses. Comparisons on bacterial levels among the different infants’ groups were achieved by using a *t*-test with Bonferroni’s correction. Multiple mixed linear models were used for studying the effect of the microbial levels at one month and the weight gain, weight, height and Z-scores at 1, 6 and 12 months. These models were used unadjusted and after adjusting for potential confounders in both groups of infants (vaginally or C-section-delivered). Backward stepwise analyses based on the Aikaike Information Criterion (AIC) were employed to determine whether the variables were included in a potential predictive model. A forest plot was used to show the effect sizes (with 95% confidence intervals) in both the so-labeled univariate and the multivariate models in both groups of infants (adjusting by infant gender and feeding type). Time variation in weight was determined according to the tertile classification of each of the microbial groups analyzed in this study. For this purpose, the cut-off points established were: 1) for vaginally delivered babies; *Bacteroides* group (T1 < 6.72; T2 6.72–8.59; T3 > 8.59); *Bifidobacterium* (T1 < 8.37; T2 8.37–8.98; T3 > 8.98); Enterobacteriaceae (T1 < 7.79; T2 7.79–8.60; T3 > 8.60); *Enterococcus* (T1 < 6.52, T2 6.52–7.60, T3 >7.60); *Lactobacillus* group (T1 < 5.60, T2 5.60–6.75, T3 > 6.75); *Staphylococcus* (T1 < 5.94, T2 5.94–6.75, T3 > 6.75) and 2) for C-section-delivered babies; *Bacteroides* group (T1 < 6.48; T2 6.48–7.30; T3 > 7.30); *Bifidobacterium* (T1 < 7.78; T2 7.78–8.79; T3 > 8.79); Enterobacteriaceae (T1 < 6.95; T2 6.95–8.42; T3 > 8.42); *Enterococcus* (T1 < 6.58, T2 6.58−7.92, T3 > 7.92); *Lactobacillus* group (T1 < 5.23, T2 5.23−6.54, T3 > 6.54); *Staphylococcus* (T1 < 5.40, T2 5.40–6.77, T3 > 6.77). *p*-values below 0.05 were considered statistically significant.

## 3. Results

### 3.1. General Description of the Population

The 124 full-term babies (55 males/69 females) included in this study were born at gestational ages ranging from 37 to 41 weeks (mean 39.6). Of these, 88 babies were delivered vaginally (birth weights between 2135 and 4800 g) and 36 by C-section (birth weights between 2215 and 4690 g). There were no statistically significant differences in mean weight according to delivery mode (mean weight of 3189 vs. 3215 for vaginal and C-section babies, respectively). Fifty-six of the infants born vaginally were exclusively breastfed, whereas 31 babies received formula or mixed feeding at the age of 1 month. In C-section babies, the proportion of children receiving each of these feeding types was 50 percent. Female babies showed a significantly higher rate of vaginal delivery than males (80% vs. 58%, *p* = 0.019), whereas no differences in feeding habits were observed between boys and girls.

### 3.2. Gut Microbiota Composition and Weight Gain Are Affected by Different Variables

In this study, the main microbial phyla representatives were quantified by using group-specific qPCR methods. As expected, the microbiota of vaginally delivered babies was different from that of C-section ones, with significantly (*P* < 0.05) higher levels of *Bacteroides*-group of microorganisms (7.65 ± 1.42 vs. 6.74 ± 0.98 Log nº cells/g, respectively) and *Bifidobacterium* (8.52 ± 0.76 vs. 8.05 ± 1.02) in the former group. No differences between both groups of infants were observed for any of the other microbial groups analyzed (Enterobacteriaceae, 8.07 ± 1.11 vs. 7.66 ± 1.38; *Enterococcus*, 6.96 ± 1.38 vs. 6.97 ± 1.56; *Lactobacillus*, 6.08 ± 1.52 vs. 5.79 ± 1.58; *Staphylococcus*, 5.94 ± 1.56 vs. 5.84 ± 1.60). These differences in the levels of some of the microbial groups analyzed between both delivery mode groups prompted us to consider them as two different groups and analyze them separately.

In both groups of 1 month-old infants, the genus *Bifidobacterium* was the bacterial group showing the highest levels, followed by members of the Enterobacteriaceae family and *Bacteroides*-group (Table 2). Interestingly, no differences in bacterial levels were observed between 1 month-old males and females neither in vaginally delivered nor in C-section-delivered babies. Concerning infant feeding practices, exclusive breastfeeding was found to be associated with reduced levels of enterococci at 1 month of age compared to formula/mixed feeding; the differences reaching statistical significance (*P* < 0.05) in vaginally delivered babies (Table 2).

As expected, when analyzing the anthropometric parameters in the sample (Table 3), statistically significant differences were found between both genders, with body weight and height being higher in males. Moreover, C-section-delivered infants on formula/mixed-feeding showed a significantly lower birth weight and weight and height by the age of 1 month (*P* = 0.022) without noticing statistically significant differences at a later age. Z-scores showed statistically significant differences in weight for height at 1 and 6 months but not at 12 months of age, and no other statistically significant differences in Z-scores were obtained between vaginally delivered and C-section babies (Supplementary Table S1).

### 3.3. Gut Microbial Groups at 1 Month Are Associated with Weight Gain

**Figure 1:**
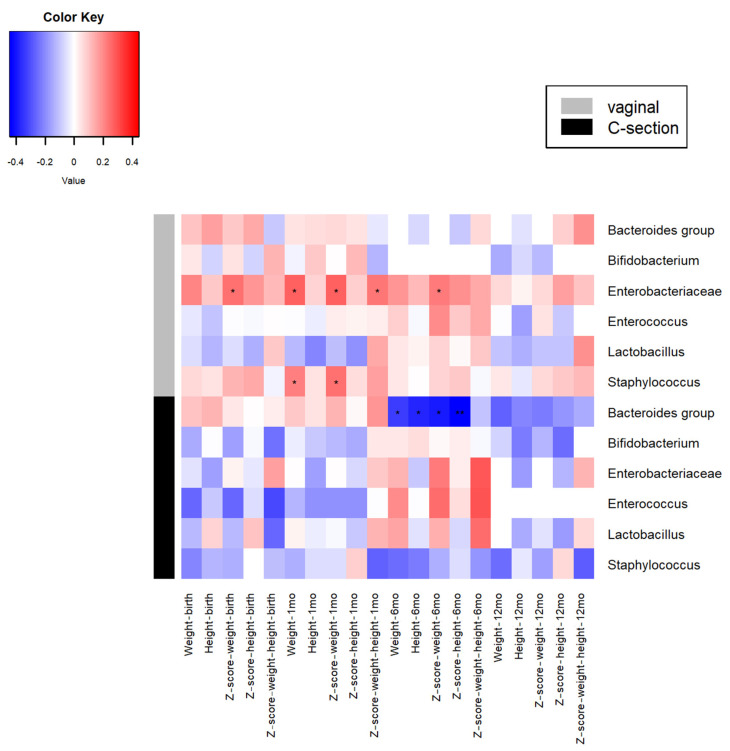
Heatmap showing the pairwise Pearson correlation coefficients between microbial groups at 1 month of age and the analyzed growth variables for both vaginally delivered and C-section-delivered babies. * *p* < 0.05.

The analysis of Pearson correlation coefficients pointed out different associations between microbes and infant growth depending on the mode of delivery (vaginal and C-section-delivered babies). In vaginally delivered infants, the family Enterobacteriaceae was the microbial group showing more correlations with the infant’s growth variables (Figure 1). A significant positive association was observed between the levels of these microorganisms at 1 month and Z-score birth weight, weight at 1 month, Z-score weight at 1 month, Z-score weight for height at 1 month and Z-score weight at 6 months (Figure 1). Similarly, in this group of infants, the levels of *Staphylococcus* showed a significant positive association with weight and Z-score for weight at 1 month of age. In C-section-delivered babies, the only significant correlations observed were the negative association between the levels of *Bacteroides* at 1 month and weight and height (as raw measures and as Z-scores) at the age of 6 months, and the direct association between levels of enterocci and weight gain at 6 months (Figure 1). Although no other statistically significant differences were obtained, the data indicate different interactions between bacteria and infant development depending on the delivery mode; the levels of some microorganisms at 1 month of age, such as *Bacteroides* or *Staphylococcus*, showed a clearly different pattern in vaginally and C-section-delivered infants.

**Figure 2:**
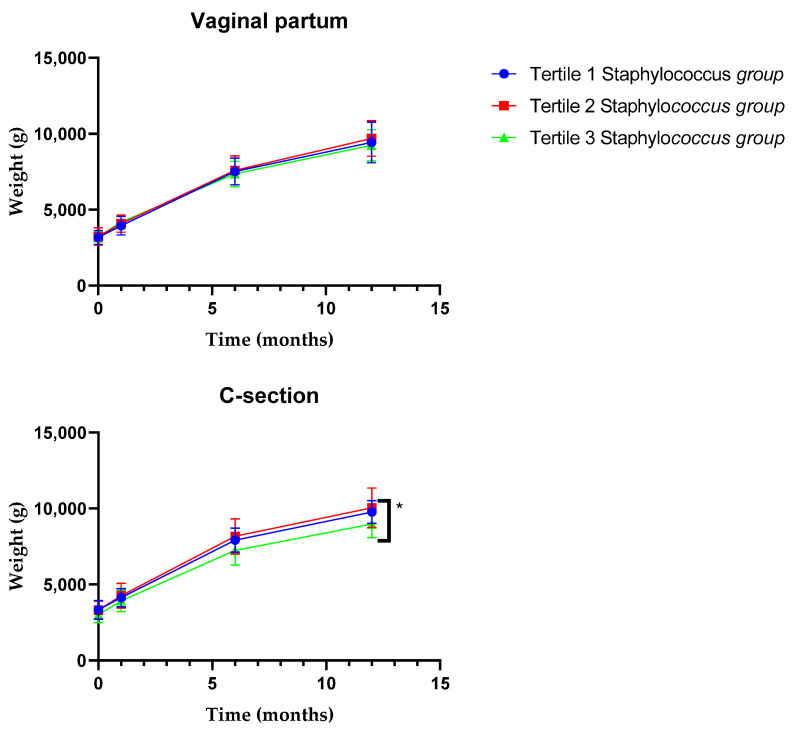
Long-term variations in body weight for the tertiles according to fecal levels of the *Staphylococcus* at 1 month of age in vaginally delivered or C-section-delivered babies (*n* = 122) (T1, tertile 1; T2, tertile 2; T3, tertile 3). * *p* ≤ 0.05.

To gain further insight into these associations, infants were classified according to the tertiles of the levels of the different microorganisms analyzed, and the variations on body weight, along the first year of life, were compared among these tertiles (Figure 2). No statistically significant differences were observed on the evolution of weight during the first 12 months of life among the tertiles for the fecal levels of *Bacteroides*, *Bifidobacterium*, Enterobacteriaceae, *Enterococcus* and *Lactobacillus*, neither in vaginally delivered nor in C-section babies. However, C-section children classified according to the tertiles obtained for the levels of *Staphylococcus* showed statistically significant differences in their weight trajectory (Figure 2). C-section infants harboring high levels of staphylococci at 1 month of age (upper tertile) displayed a significantly lower weight at 1 year of age, with this phenomenon not being observed in vaginally delivered babies.

**Figure 3:**
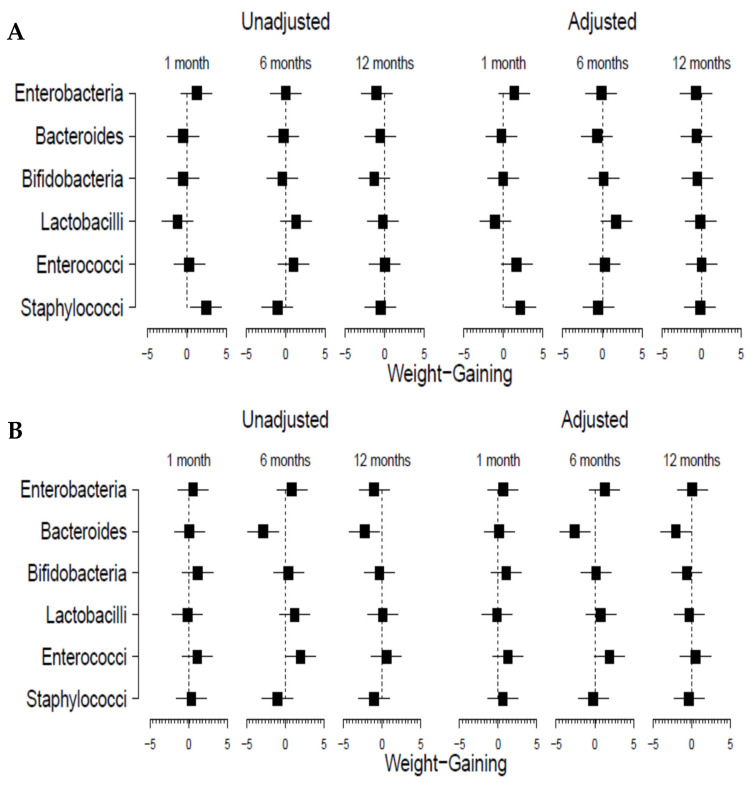
Forrest plots showing the effect sizes (with 95% confidence interval) of the association of microbiota-related variables with infant weight gain at 1, 6 and 12 months of age according to delivery mode (vaginal delivery (**A**) and C-section (**B**)). Results from unadjusted model and adjusted by gender and type of feeding. Dotted lines represent the zero value, with values on the left side indicating negative associations and those on the right side indicating associations with positive sign.

Hereafter, uni- and multivariate regression models were performed for a deeper assessment of the association between early microbiota and weigh-gain in both groups of infants (Figure 3). To take into consideration the potential effects of gender and feeding type, the models were controlled for these two variables, and the relationship between microbiota at 1 month of age and infant weight gain at 1, 6 and 12 months of age was assessed (Figure 3). Different effects were observed between both groups of infants. A significant positive effect of the levels of *Staphylococcus* at 1 month of age on weight gain at 1 month was obtained in both the unadjusted (*p* = 0.016) and adjusted (*p* = 0.036) models in vaginally delivered babies, but these do not reach significance in C-section-delivered infants. On the contrary, a negative association of *Bacteroides* levels at 1 month of age with weight gain at 6 and 12 months was observed in the C-section group (*p* = 0.007 in unadjusted and *p* = 0.014 in the adjusted model at 6 months of age, and *p* = 0.031 and *p* = 0.052, respectively, at 12 months of age). The other microbial groups analyzed did not show any statistically significant effect.

## 4. Discussion

The levels of the different microbial groups analyzed in this study were in line with those previously described for 1 month-old full-term infants [26,34,35]. Additionally, in accordance with previous studies, bifidobacteria was the bacterial group showing the highest levels, followed by enterobacteria, which is another of the dominant microbial groups in un-weaned infants [26,34]. Additionally, as expected [1], differences in the microbial levels were observed between vaginal and C-section-delivered babies.

Gender-associated differences in the infant microbiota composition and diversity have been previously reported [36,37]; however, in the present work, we did not notice any significant differences in the levels of the analyzed microbial groups between males and females, neither in vaginally nor in C-section-delivered babies. This is one aspect that deserves further attention since understanding the potential gender differences in the microbiome, the so-called microgenderome [38], and the role that these play in the risk of disease is of utmost importance for developing microbiota modulation strategies in early life. It must be taken into consideration that early life constitutes a critical moment. Some studies have reported an association between antibiotic treatments during the postnatal period microbiota [34,39] and an increased risk of obesity and related metabolic disorders [40,41]. This suggests a possible influence of microbiota alterations during this period in obesity risk later in life, as it has been demonstrated in animal models [42].

Moreover, some studies have also reported associations between early microbiota and weight gain [24,37,43]. However, due to the growing evidence linking the microbiota in early life to obesity risk, we consider that studies focused on full-term infants, as the present one, are especially relevant in this context. In this regard, previous studies demonstrated an altered microbiota during the first year of life in infants developing obesity later on [20], pointing out at the first months of life as the key moment for later metabolic homeostasis.

Interestingly, some microorganisms, such as *Staphylococcus* or *Enterococcus*, have been previously reported to be negatively associated with infant weight/weight gain in preterm infants during very early life [24]. The levels of these microorganisms were also found to be lower, at 5 and 9 months of life, in excessive weight gaining full-term infants than in those showing an appropriate weight gain [43]. However, some differences among studies are also present, likely due to the different methodologies and experimental designs used; for instance, we analyzed the microbiota at 1 month of age, whereas others analyzed it at a later stage (5 and 9 months of age) [43], and we segregated the analyses by delivery mode whilst other authors did not. Actually, our results indicate the existence of different interactions in vaginally delivered and in C-section-delivered babies. We observed that changes in the sign of the microbe-weigh association might occur along different sampling times, as evidenced by our data on staphylococci, showing a positive association with weight gain at 1 month of age but not at later ages when the interaction seems to be even negative. Interestingly, in C-section-delivered babies, but not in vaginal infants, the levels of the *Bacteroides*-group at 1 month of age correlated negatively with later weight, even when the model was adjusted by feeding mode. Delayed colonization by this microorganism has been often reported in C-section-delivered babies [44,45] and C-section delivery has been linked to increased risk of childhood obesity [46]. These observations point out at the levels of *Bacteroides* during early life as a potential early marker for the later risk of excessive weight gain in C-section-delivered babies, an aspect that should be the subject of further studies.

It is important to point out that different factors may influence infant growth trajectories. Among these, infant feeding habits may be of importance. Our results showed that exclusive breastfeeding was associated with significantly lower levels of *Enterococcus* in vaginally delivered babies and with a trend (non-statistically significant) also observed in the C-section-delivered group. In the former group, a trend towards higher *Staphylococcus* levels was also observed. These two microorganisms have been linked to the feeding pattern of the infant. Breastmilk has been previously described as a source of *Staphylococcus*, with increased levels of this microorganism being found in breastfed babies [47]. Other studies, in accordance with our results, reported lower levels of *Enterococcus* in breastfed infants [48]. Altogether, these results suggest that the observed differences in microbial groups and weight gain may be partly related to the feeding habit of the infant. However, although the feeding habit is likely an important factor, our multivariate models were corrected for this variable and some of the effects still remained significant, indicating a microbiota–weight association independent of the feeding type. Therefore, the microbiota–host relation needs to be considered in the analyses focused on infant growth trajectories in order to shed light on the influence of this relationship for child development. Once this relationship is fully understood, it may be possible to develop nutritional strategies, such as dietary probiotics or prebiotics targeting the infant, or perhaps the pregnant or lactating mother, for modulating early life microbiota and the later infant weight gain.

It is also important to underline that our sample size is still limited for establishing strong general conclusions, especially in a context where several potential confounder factors may be present, as is the case in infant microbiota studies. However, it is also true that the infants included originated not just from a unique hospital and geographical location, which could be a source of bias, but from two distant locations. It is worth pointing out as well that our microbiota data are restricted to defined microbial groups for which absolute levels were determined and the potential influence of other microorganisms may have been overseen.

## 5. Conclusions

This work is among the first ones assessing the relationship between the absolute levels of relevant early life intestinal microorganisms, such as bifidobacteria, enterobacteria, lactobacilli, enterococci or staphylococci, and the later weight gain in either vaginally or C-section-delivered full-term infants. The data point out the relationship between specific infant gut microbes and healthy infant development. Our results underline the interest in exploring the intestinal microbiota as a potential target for favoring proper growth and weight gain in infants with potential consequences in later health.

## Figures and Tables

**Table 1:** Primers and PCR conditions for the different primer pairs used in this study.

Bacterial Group	Primers	Tm	Reference
*Bacteroides*-group	F-GAGAGGAAGGTCCCCCAC R-CGCKACTTGGCTGGTTCAG	56·°C	[26]
*Bifidobacterium* genus	F-GATTCTGGCTCAGGATGAACGC R-CTGATAGGACGCGACCCCAT	60 °C	[27]
Enterobacteriaceae family	F-CATTGACGTTACCCGCAGAAGAAGC R-CTCTACGAGACTCAAGCTTGC	63 °C	[28]
*Enterococcus* genus	F-CCCTTATTGTTAGTTGCCATCATT R-ACTCGTTGTACTTCCCATTGT	61 °C	[29]
*Lactobacillus*-group	F-AGCAGTAGGGAATCTTCCA R-CACCGCTACACATGGAG	58 °C	[30,31]
*Staphylococcus* genus	F-ACGGTCTTGCTGTCACTTATA R-TACACATATGTTCTTCCCTAATAA	56 °C	[32]

**Table 2:** Levels (Log nº cells/g) of some relevant bacterial groups in fecal samples of the vaginally delivered or C-section-delivered infant population included in this study, categorized by feeding type, gender and mode of delivery.

		Vaginally Delivered Babies (*n* = 88)				C-Section-Delivered Babies (*n* = 36)			
	Bacterial Groups	Gender		Feeding Type		Gender		Feeding Type	
Phyla		Male	Female	EBF	MF	Male	Female	EBF	MF
		(*n* = 33)	(*n* = 55)	(*n* = 56)	(*n* = 31)	(*n* = 22)	(*n* = 14)	(*n* = 18)	(*n* = 18)
Bacteroidetes	*Bacteroides* group	7.63 ± 1.57 ^$^	7.67 ± 1.34 ^$^	7.55 ± 1.57	7.79 ± 1.08 ^$^	6.67 ± 0.97 ^$^	6.85 ± 1.03 ^$^	6.85 ± 0.88	6.63 ± 1.09 ^$^
Actinobacteria	*Bifidobacterium* genus	8.45 ± 0.92	8.57 ± 0.65 ^$^	8.48 ± 0.76	8.60 ± 0.78 ^$^	8.16 ± 1.01	7.89 ± 1.06 ^$^	8.05 ± 1.10	8.05 ± 0.98 ^$^
Proteobacteria	Enterobacteriaceae	8.18 ± 1.11	8.01 ± 1.12	8.01 ± 1.00	8.17 ± 1.33	7.51 ± 1.38	7.89 ± 1.39	7.69 ± 1.31	7.62 ± 1.48
Firmicutes	*Enterococcus* genus	6.64 ± 1.64	7.15 ± 1.16	6.54 ± 1.31 *	7.77 ± 1.13 *	6.89 ± 1.38	7.10 ± 1.87	6.60 ± 1.68	7.34 ± 1.38
	*Lactobacillus* group	6.05 ± 1.63	6.10 ± 1.47	6.08 ± 1.55	6.10 ± 1.52	5.95 ± 1.63	5.55 ± 1.52	5.41 ± 1.66	6.17 ± 1.44
	*Staphylococcus* genus	6.00 ± 1.48	6.02 ± 1.41	6.15 ± 1.47	5.71 ± 1.34	5.43 ± 1.55	6.48 ± 1.51	5.98 ± 1.62	5.70 ± 1.61

All values are shown as mean ± standard deviation. EBF, exclusive breastfeeding; MF, formula/mixed feeding. There is a missing value in feeding type (*n* = 87). * Denotes statistically significant differences (*p* ≤ 0.05) between genders or feeding types within the same delivery group. ^$^ Denotes statistically significant differences (*p* ≤ 0.05) for infants from the same gender or feeding type between the two delivery groups (vaginally delivered or C-section-delivered).

**Table 3:** Weight and weight gain during the first year of life in the infants included in this study as categorized by feeding type, gender and delivery mode.

	Vaginally Delivered Babies (*n* = 88)				C-Section-Delivered Babies (*n* = 36)			
	Gender		Feeding Type		Gender		Feeding Type	
	Male	Female	EBF	MF	Male	Female	EBF	MF
Variable	(*n* = 33)	(*n* = 55)	(*n* = 56)	(*n* = 31)	(*n* = 22)	(*n* = 14)	(*n* = 18)	(*n* = 18)
Weight birth (g)	3358 ± 520 *	3036 ± 579 *	3202 ± 328	3087 ± 867	3317 ± 629	3055 ± 479	3504 ± 479 *	2927 ± 508 *
Height birth (cm)	49.9 ± 2.6	48.7 ± 1.8	49.2 ± 1.8	48.9 ± 2.9	49.7 ± 2.6	48.6 ± 2.1	50.0 ± 2.3	48.5 ± 2.4
Weight 1 month (g)	4310 ± 531 *	3898 ± 497 *	4117 ± 428 ^$^	3954 ± 702	4235 ± 751	3887 ± 602	4415 ± 769 *^,$^	3784 ± 482 *
Height 1 month (cm)	54.1 ± 2.0	53.2 ± 2.7	53.7 ± 2.2	53.3 ± 3.1	53.5 ± 2.8	52.6 ± 2.8	54.6 ± 2.2 *	51.7 ± 2.6 *
Weight 6 month (g)	8129 ± 712 *	7106 ± 738 *	7355 ± 860	7721 ± 877	8142 ± 972 *	7206 ± 890 *	7951 ± 1169	7606 ± 886
Height 6 month (cm)	68.4 ± 2.3 *	65.9 ± 2.5 *	66.6 ± 2.8	67.2 ± 2.6	67.4 ± 2.5 *	65.7 ± 2.5 *	67.2 ± 2.5	66.3 ± 2.7
Weight 12 month (g)	10369 ± 1003 *	8921 ± 921 *	9414 ± 1204	9515 ± 1142	9971 ± 1019 *	9020 ± 937 *	9838 ± 1227	9363 ± 885
Height 12 month (cm)	76.7 ± 2.9 *	73.5 ± 2.9 *	74.9 ± 3.3	74.4 ± 3.3	75.3 ± 2.5	73.5 ± 3.8	75.5 ± 2.8	73.7 ± 3.4
Weight gain 1 month (g)	952 ± 305	872 ± 511	914 ± 334	884 ± 609	917 ± 344	832 ± 292	911 ± 397	857 ± 235
Weight gain 6 month (g)	4717 ± 633 *	4071 ± 876 *	4208 ± 882	4499 ± 767	4824 ± 1058 *	4151 ± 746 *	4446 ± 905	4679 ± 1091
Weight gain 12 month (g)	7015 ± 882 *	5886 ± 1030 *	6271 ± 1200	6350 ± 950	6653 ± 1026 *	5965 ± 831 *	6334 ± 1007	6437 ± 1024

All values are shown as mean ± standard deviation. EBF, exclusive breastfeeding; MF, formula/mixed feeding. * Denotes statistically significant differences (*p* ≤ 0.05) between gender or feeding types within the same delivery group. ^$^ Denotes statistically significant differences (*p* ≤ 0.05) for infants from the same gender or feeding type between the two delivery groups (vaginally delivered or C-section-delivered).

## Data Availability

The data are available upon reasoned request to the authors, under the restrictions established by the ethical approval of the study.

## Supplementary Materials

The following are available online at https://www.mdpi.com/article/10.3390/nu13072412/s1, Table S1: General description of the WHO Z-scores in the sample across time by type of partum and feeding.
