# Supplementary material for: Levels of Predominant Intestinal Microorganisms in 1 Month-Old Full-Term Babies and Weight Gain during the First Year of Life
Source: Nutrients. 2021 Jul 14;13(7):2412. doi: 10.3390/nu13072412 (PMC8308764; doi:10.3390/nu13072412)
Supplement: Supplementary file 1 [file nutrients-13-02412-s001.zip › nutrients-1280529-supplementary.pdf]

Table S1. General description of the WHO z-scores in the sample across time by type of partum and feeding.

				Type of delivery	
	**Median**	**P25**	**P 75**	**Vaginal**	**C-section**
z-score_weight birth	-0.46	-1.12	0.31	-0.37±0.95	-0.39 ±1.22
z-score height birth	-0.44	-1.23	0.46	-0.44 ± 1.16	-0.45 ± 1.27
z-score_weight/height birth	-0.20	-0.77	0.46	-0.13 ± -0.95	-0.19 ± 1.22
z-score weight 1 mo	-0.42	-1.16	0.22	-0.47 ± 0.93	-.049 ± 1.18
z-score height 1 mo	-0.32	-1.17	0.44	-0.25 ± 1.27	-0.62 ± 1.39
z-score weight/height 1 mo	-0.26	-0.96	0.52	-0.44 ± 1.32	0.06 ± 1.07*
z-score weight 6 mo	-0.16	-0.83	0.31	-0.24 ± 0.93	-0.009 ± 1.08
z-score height 6 mo	-0.11	-0.97	0.70	-0.06 ± 1.16	-0.29 ± 1.26
z-score weight/height 6 mo	-0.13	-0.61	0.52	-0.19 ± 0.78	0.20 ± 1.00*
z-score weight 12 mo	0.14	-0.42	0.65	0.18 ± 0.91	0.17 ± 0.89
z-score height 12 mo	0.00	-0.78	0.76	-0.22 ± 2.62	-0.17 ± 1.23
z-score weight/height 12 mo	0.200	-0.390	0.860	0.67 ± 4.63	0.35 ± 0.92

P25, percentile 25; P75, percentile 75. EBF. Exclusive breastfeeding; MF: mixed feeding; mo: month. *p ≤0.05.
